# Cutaneous metastasis of uterine carcinosarcoma mimicking drug eruption

**DOI:** 10.1016/j.jdcr.2024.03.006

**Published:** 2024-03-19

**Authors:** Katherine L. Wang, Olivia M. Crum, Amy A. Swanson, Emma F. Johnson

**Affiliations:** a Mayo Clinic Alix School of Medicine, Mayo Clinic, Jacksonville, Florida; b Department of Dermatology, Mayo Clinic, Rochester, Minnesota; c Department of Laboratory Medicine and Pathology, Mayo Clinic, Rochester, Minnesota

**Keywords:** carcinosarcoma, cutaneous metastasis, doxorubicin, drug eruption, endometrial neoplasm

## Introduction

Uterine carcinosarcoma (UCS), also known as malignant mixed Müllerian tumor, is a rare and aggressive cancer of the uterus primarily affecting women aged 60 and older. It contains both carcinomatous and sarcomatous elements, with the sarcomatous component widely considered to be dedifferentiated from an initial carcinomatous progenitor.^1^ It affects 1.36/100,000 women and is associated with a poor prognosis, with 30% to 40% of patients presenting with extrauterine disease at time of diagnosis. Although it represents less than 5% of all uterine malignancies, it is responsible for 15% of deaths from uterine cancer, with an overall 5-year survival rate of less than 40%.^2^

Metastasis is common, with a predilection for lymph nodes and intraabdominal sites such as the ovaries, fallopian tubes, and omentum. However, cutaneous involvement is rare, representing only 1.3% of metastases in a 2004 study by Callister et al.^3^ Few cases have been reported in the literature. We present a case of metastatic UCS to the skin clinically mimicking a drug eruption.

## Case report

A 71-year-old Caucasian woman with Fitzpatrick II skin type presented to the dermatology clinic for evaluation of a nonpruritic rash that had been present for a few months. She was referred by oncology for suspicion of a drug rash secondary to doxorubicin, which she had been taking for 10 months. The rash originated on her left thigh before expanding to involve the right thigh, abdomen, and vulvar area. Previous treatments included oral prednisone, topical mupirocin, and emollients, with no improvement.

Her history was significant for UCS initially diagnosed 5 years prior. The primary mass was surgically removed with total abdominal hysterectomy, bilateral salpingo-oophorectomy, and bilateral pelvic lymphadenectomy revealing negative nodes. This was followed by a course of radiation to the endometrium. Unfortunately, she was found to have progressive disease a year later and received several rounds of chemotherapy and immunotherapy, including doxorubicin/bevacizumab, which she had been on for approximately 1 year prior to presentation to the dermatology clinic. Shortly before her presentation to the dermatology clinic, doxorubicin was discontinued due to concerns that her rash was drug related.

**Fig 1:**
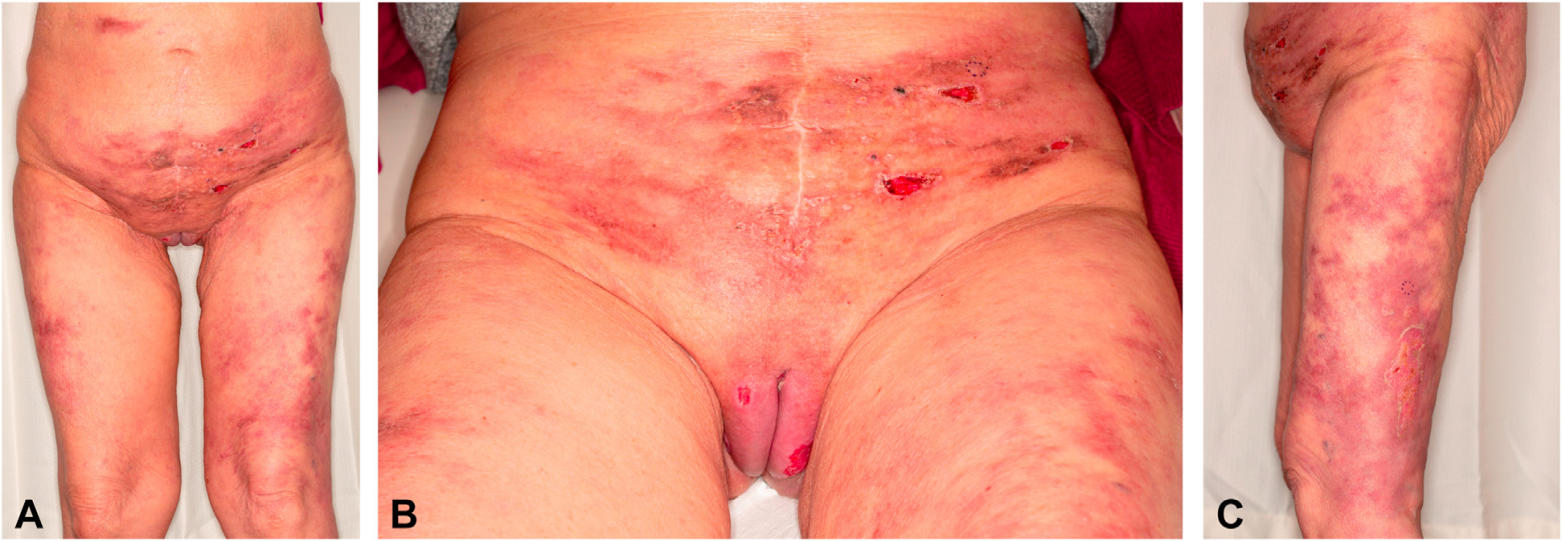
Clinical presentation of metastatic uterine carcinosarcoma to the skin. **A,** Persistent, nontender, indurated, erythematous rash with interspersed hyperpigmented patches involving the abdomen and bilateral thighs. **B,** Areas of ulceration are present over a violaceous edematous base on the vulva. **C,** Reticular rash with areas of skin breakdown on the left lateral thigh.

Physical examination revealed indurated erythematous papules and plaques involving the lower abdomen (Fig 1, *A*), external genitalia (Fig 1, *B*), buttock, and proximal thighs (Fig 1, *C*), some of which had skin breakdown and shallow well-demarcated ulcerations. No palmoplantar lesions were noted. Based on the clinical history and physical exam, several etiologies were considered, including drug-related rash, reactive granulomatous dermatitis, infection, calciphylaxis, vasculitis, vasculopathy, cutaneous T-cell lymphoma, and cutaneous metastasis. In addition to bacterial and fungal cultures, punch biopsies were obtained from the abdomen and left thigh.

**Fig 2:**
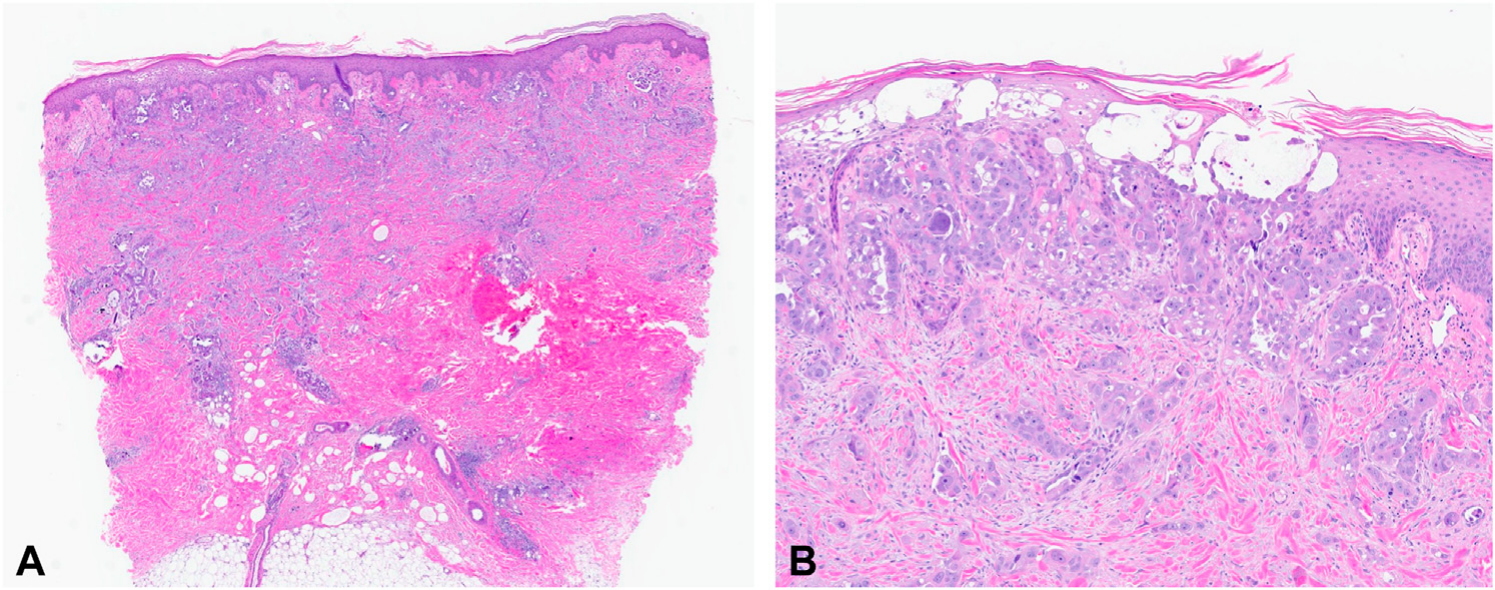
Cutaneous involvement by metastatic uterine carcinosarcoma. **A,** Punch biopsy demonstrating diffuse infiltration of the dermis with atypical epithelioid cells. **B,** Atypical, large, epithelioid cells with large irregular nuclei and prominent nucleoli demonstrating an infiltrative growth pattern, epitheliotropism, and foci of lymphovascular invasion. (**A** and **B,** Hematoxylin-eosin stain; original magnifications: **A,** 2×; **B,** 10×.)

Punch biopsy specimens demonstrated diffuse infiltration of atypical epithelioid cells (Fig 2, *A*) with abundant cytoplasm, large pleomorphic nuclei, and prominent nucleoli (Fig 2, *B*). Numerous areas of lymphovascular invasion and epidermotropism were noted. Immunohistochemical staining demonstrated strong and diffuse positivity of the neoplastic cells for paired box gene 8 and tumor protein p53. These findings were consistent with metastasis from the patient’s UCS.

## Discussion

This case highlights the importance of considering skin metastasis when evaluating a rash in a patient with a history of cancer. Special attention should be given if the rash is indurated, persistent, or recalcitrant to standard therapy. While the classic presentation of cutaneous metastases is a dermal nodule, clinicians should maintain a high index of suspicion in such patients and keep skin metastasis in their differential regardless of lesion morphology.

In general, cutaneous metastases are uncommon, occurring in 1% to 10% of cancer patients.^4^ Cutaneous metastases represent an uncommon, deadly, and late-developing complication in most patients. Studies regarding frequency of metastases have varying results given inherent biases in retrospective reviews with regard to patient population and primary malignancies. It is generally accepted that in women, breast cancer, colon cancer, and melanoma represent the most frequent primary tumors, while in men, lung cancer, colon cancer, and melanoma are the most common.^5^ The prevalence of cutaneous metastases from endometrial cancer has been reported at about 0.8%.^6^

UCS is classified as a high-risk endometrial carcinoma, arising from a malignant epithelial clone that subsequently dedifferentiates to form the sarcomatous element of this tumor. It is composed of various possible mixtures of carcinomatous and sarcomatous components—our patient’s primary tumor consisted of 80% sarcoma and 20% carcinoma. UCS demonstrates a predilection for lymphatic spread, and positive extrauterine nodes are common at the time of presentation.

The carcinomatous element of UCS is noted to be highly aggressive and play a dominant role in metastasis^7^; indeed, our patient’s cutaneous metastases displayed only epithelial elements. Clairwood et al described a case of metastatic UCS presenting as firm fixed nodules on the cheek, back, and flank.^8^ Intriguingly, the metastatic lesion in this case was comprised solely of sarcomatous elements. Cho et al previously reported a case of metastatic UCS to the scalp in a 43-year-old woman presenting as a solitary, painless, hard papule that developed over 2 to 3 months.^9^ Biopsy of the lesion demonstrated predominant sarcomatous elements. This illustrates the varied presentations of cutaneous metastases in primary UCS and emphasizes the importance of prompt identification in guiding further management.

In conclusion, we present this case of rare cutaneous metastases from UCS to illustrate the varied clinical morphology that cutaneous metastases can display. It is important to keep cutaneous metastases on the clinical and pathologic differential diagnoses, despite their rarity. Biopsy and histopathologic review remain critical diagnostic tools in the diagnosis of cutaneous metastases.

## Conflicts of interest

None disclosed.
